# 定位技术在电视胸腔镜诊治孤立性肺小结节中的应用

**DOI:** 10.3779/j.issn.1009-3419.2012.02.07

**Published:** 2012-02-20

**Authors:** 剑飞 叶, 宏宇 白, 圆 王, 旭峰 刘, 伟 辛, 洪刚 夏, 军 陈

**Affiliations:** 1 300280 天津，天津市大港油田总医院胸外科 Department of Thoracic Surgery, Dagang Oil Field General Hospital, Tianjin 300280, China; 2 300052 天津，天津医科大学总医院肺外科，天津市肺癌研究所 Department of Lung Cancer Surgery, Tianjin Medical University General Hospital, Tianjin Lung Cancer Institute, Tianjin 300052, China

**Keywords:** 孤立性肺结节, 电视胸腔镜手术, 定位技术, Solitary pulmonary nodule (SPN), Video-assistant thoracoscopic surgery, Localization technique

## Abstract

**背景与目的:**

电视胸腔镜已广泛应用于孤立性肺小结节（solitary pulmonary nodule, SPN）的诊治，但经常遇到术中无法准确定位病灶的情况。本研究探讨电视胸腔镜手术（video-assistant thoracoscopic surgery, VATS）在诊治孤立性肺小结节过程中的定位技术的临床应用价值。

**方法:**

对孤立性肺小结节患者，术前先行三维CT定位，如SPN≤1.5 cm，或深达肺胸膜下2.0 cm，予行Hook-wire定位，术中先用器械间接触诊法，如失败改行电视胸腔镜辅助小切口（video-assisted minithoracotomy, VAMS），用手指直接触诊法定位，术中定位成功后，以直线切割缝合器行SPN楔形切除术，术后快速冰冻病理检查以决定下一步治疗方案。

**结果:**

本组23例完成了胸腔镜SPN的切除，其中完全胸腔镜手术10例，VAMS 13例。

**结论:**

电视胸腔镜SPN切除术术前术中的精确定位具有临床应用价值。

## Abstract (en)

**Background and objective:**

Video-assisted thoracoscopy is widely used in diagnosis and treatment of solitary pulmonary nodule (SPN). However, localization of the nodule during surgery is occasionally difficult. The purpose of the current research is to evaluate the localization technique during diagnosis and treatment of SPN with video-assistant thoracoscopic surgery (VATS).

**Methods:**

Three-dimensional computed tomography is performed for SPN localization. If SPN is less than 1.5 cm or more than 2.0 cm beneath the pleura, Hook-wire localization technique is used. During operation, initial action is palpation with equipment. If this method fails, video-assisted minithoracotomy (VAMS) and direct finger use are applied for localization. Upon successful localization, a linear cutter is used for wedge resectioning. Fast frozen pathology is employed to make the decision for the next procedure.

**Results:**

SPNs are resected for 23 cases. Among them, 10 cases are performed with VATS and 13 cases are conducted with VAMS.

**Conclusion:**

The accurate localization of SPN before and during surgery is very significant for the resectioning of SPN by VATS.

随着现代科技进步、人民生活水平的不断提高，螺旋CT已经日益成为胸部体检的一项常规检查项目，这使得肺部病变的发现变得越来越早，越来越容易，需要外科干预的孤立性肺小结节（solitary pulmonary nodule, SPN）的检出率也越来越高。鉴于临床发现的SPN近半数为恶性，因此需积极采用外科手术处理^[1]^。研究表明^[2]^，在SPN的诊断方面，胸腔镜肺结节楔形切除术可达到与开胸手术同样的效果。但是在胸腔镜手术中经常会出现寻找不到病灶的情况，即使扩大切口，改用手指触诊法寻找病灶，对于直径较小的SPN也无能为力。为此，天津市大港油田总医院胸外科于2006年引进了Hook-wire术前定位结合术中定位及术中快速冰冻活检的方法，获得了满意效果，现报道如下。

## 1 资料与方法

### 1.1 临床资料

本组26例，均为胸部CT发现SPN，结节≤1.5 cm，或深达肺胸膜下2.0 cm以上，或为毛玻璃样病变，且距离大血管3.0 cm以上的患者。其中男17例，女9例；年龄42岁-80岁，平均59.1岁。吸烟者15例，不吸烟者11例。病变位于左肺上叶8例，左肺下叶2例，右肺上叶9例，右肺中叶3例，右肺下叶4例。

### 1.2 术前Hook-wire定位方法

根据患者的影像学资料，明确病灶所在部位，并在CT指导下行Hook-wire定位。根据“垂直就近”的原则，选择进针部位，确定进针深度及角度，常规消毒、铺巾，穿刺点局麻后，使用Hook-wire定位系统套针穿刺，穿刺完成后，再次行CT扫描，如显示倒钩膨胀打开，且在结节附近10 mm以内，即判定穿刺成功，然后紧贴胸壁剪断钢丝。随后在患侧胸壁锁骨中线第2肋间消毒、铺巾、穿刺，胸腔内置入14#带侧孔中心静脉导管，接引流袋引流，患者即送手术室行胸腔镜手术。

### 1.3 麻醉方法

采用双腔管全身麻醉。

### 1.4 手术方法

麻醉成功后，去除引流袋，行胸腔镜手术。胸腔镜置入后，术中在患肺完全萎陷的情况下，抓起定位钢丝，结合3维重建图像，以肺钳轻柔钳夹病变部位，确定部位后，以直线切割缝合器楔形切除病变。如单纯胸腔镜下不能确定病灶，则根据病灶部位，选择最适部位切小口，以可置入一食指为度，伸入食指进行触摸Hook-wire倒钩所在部位，待确定后，仍以直线切割缝合器楔形切除病变。切除完毕后，使用取物袋将标本自胸壁切口取出；将标本送快速冰冻活检，以确定是否取到病灶，并确定是否需要进一步治疗，如发现为恶性肿瘤，即行肺叶切除及纵隔淋巴结清扫的规范治疗。手术结束后，决定是否拔除于定位时置入胸腔之中心静脉导管。

## 2 结果

本组26例中有3例因胸腔内粘连紧密，改行开胸法肺楔形切除术，其余23例在胸腔镜下完成了SPN的切除，有10例术中通过肺钳间接触诊法确定病灶并行完全胸腔镜SPN切除，13例使用手指直接触诊法定位，行VAMS切除SPN。术后病理证实良性病变12例（46.15%）：其中肺结核3例，错构瘤3例，炎性假瘤4例，机化性纤维化结节2例；恶性病变14例（53.85%），其中腺癌6例，鳞癌5例，小细胞肺癌3例。10例良性者随即结束手术，14例恶性肿瘤中3例因肺功能差不能耐受较大手术，未做进一步处理，10例行标准肺叶切除+纵隔淋巴结清扫术。在全部26例患者中有7例发生气胸，5例发生穿刺局部血肿，3例导丝移位，并发症发生率57.6%，但均未导致手术失败。

## 3 讨论

SPN是指单一的、边界清楚的、影像不透明的、直径≤3 cm、周围为含气肺组织所包绕，不伴肺不张、淋巴结转移或胸腔积液表现的肺部结节。据统计，恶性率可以达到73.6%^[3]^，本组恶性率为53.85%。因临床发现的SPN有大约近半数为恶性，而且部分肺癌已不是早期，所以均应积极采用外科手术，以达到早诊断、早治疗的目的^[1]^。Santambrogio等^[2]^的研究证明电视胸腔镜手术对SPN的诊断可达到与开胸手术同样的效果，切除准确率可达到100%，且可大大减少术后病人的痛苦，并明显缩短住院时间。但是由于电视胸腔镜手术时手术者不能像开胸手术一样直接用手全面探查病变部位，病灶定位存在一定困难^[1]^。有文献^[4]^报道用CT引导下注射亚甲蓝定位的方法，但效果并不能令人满意。也有报道^[5]^在术中使用超声探头定位的方法，其定位精确度可达100%，但开展此项技术需要专门的设备，国内尚无这方面的报道。

CT引导下Hook-wire术前定位是一种安全、有效的术前定位方法。Wicky等^[6]^报道23例患者均获得准确定位，并极力推荐此方法指导SPN的VATS手术。我们自2006年开始将Hook-wire系统应用于临床实践，获得了以下一些经验：①定位时要在定位侧置管行胸腔闭式引流，以避免在全麻插管过程中机械通气导致张力性气胸；②定位后应立即进手术室手术，麻醉要求行双腔管麻醉，便于电视胸腔镜探查及手术；③术中先用器械间接触诊法确定病灶，如失败再改行VAMS手指直接触诊法，这样可尽量做到完全胸腔镜的SPN切除活检术，如为良性病变，患者术后恢复将明显好于VAMS；④切除完毕后，用取物袋将标本自胸壁切口取出；术中一定要行快速冰冻活检，以确定是否取到病灶，并确定是否需要进一步治疗；如发现为恶性肿瘤，除非患者不能耐受肺叶切除，按常规即行肺叶切除及纵隔淋巴结清扫术^[7]^。

根据文献^[8]^报导，Hook-wire的并发症主要有气胸、Hook-wire移位、局限性血肿、气体栓塞等。本组病例并发症发生率为57.6%，但未发生气体栓塞这种造成手术失败的严重并发症，Hook-wire发生移位的病例，移位幅度也未影响手术。

总结本组手术的优点是：①最大限度地利用了电视胸腔镜微创的特点，减少了辅助小切口的应用。对于良性结节，电视胸腔镜手术在完整切除肿瘤的同时明确了诊断，不仅消除了良性结节恶变的可能，还最大限度保留了肺功能，患者术后恢复快，不会发生开胸手术后的疼痛、手术区感觉障碍等相关并发症，充分体现了微创的优点；②对于恶性结节，术前的精确定位能够做到不遗漏病灶，尽快明确诊断，使患者得到及时、合理的治疗，真正体现了肺癌早发现、早诊断、早治疗的原则；③定位时在CT引导下行胸腔闭式引流术，减少了麻醉过程中发生张力性气胸的风险；④使用取物袋取出标本，使标本在经过手术切口时不会发生恶性肿瘤的种植转移，体现了恶性肿瘤治疗过程中的无瘤原则。
